# Book Reviews

**Published:** 1918-11

**Authors:** 


### BOOK REVIEWS

Books mentioned may be inspected at and ordered through this office. So far as possible, books received in any month will be reviewed in the issue of the second month following. Pamphlets, quarterly and similar periodicals, reports, transactions, etc., will, as a rule, merely be mentioned.

---

**Review of War Surgery and Medicine**: This monthly magazine which is prepared in the office of the Surgeon General contains the latest reports of treatments of diseases occurring in the military zones. Copies may be seen in the Buffalo Grosvenor Library.

---

**The Wassermann Test.** By Charles F. Craig, A. M. (Hon.), M. D. (Yale), Lieutenant Colonel, Medical Corps, United States Army; Fellow of the American College of Surgeons; Formerly Assistant Professor of Bacteriology and Pathology, Army Medical School, and George Washington University; Commanding Officer, Department Laboratory, Central Department, United States Army, Fort Leavenworth, Kansas. Published with authority of the Surgeon General, United States Army. Cloth, 239 pages. Illustrated with Colored Plates, Halftone Plates, and Fifty-seven Tables. C. V. Mosby Co., St. Louis, 1918.

This work on the Wassermann test will be of great value to the medical profession in general, but especially will it be welcomed by those who in the past have read the numerous valuable contributions to the literature of the subject by the same writer.

Craig, by reason of his vast practical experience and the clearness and accuracy of his writings on the Wasserman test has come to be regarded as one of the highest authorities. The figures and statistics which he presents in this volume are impartially taken from large numbers of cases in the army, over whose treatment there is almost perfect control.

The principal of the Wassermann reaction is cleverly unfolded while giving the history of its discovery, which together with the schematic representations offers a lucid explanation of the phenomena of bacteriolysis and hemolysis. Whereas the author's nomenclature may seem at first confusing, it stands for simplicity and standardization, as he clearly states a positive reaction should not be termed four plus, Craig's two plus, unless the test has been done with various rated doses of complement as in the methods of Citron, and of Browning and McKenzie.

On the whole the book is very well written and clearly illustrated and will serve as a concise and authoritative exposition of the well known facts of the Wassermann reaction.

---

**War Surgery of the Abdomen.** By Cuthbert Wallace, C. M. F., F. R. C. S. Eng., M. B., B. S. Lond., Surgeon St. Thomas' Hospital; Lecturer on Surgery in the Medical School; Consulting Surgeon, British Armies in France. Cloth, 152 pages with 26 illustrations. Price, $3.00 net. Philadelphia: P. Blakiston's Son & Co., 1012 Walnut St., 1918.

Wallace has written this valuable brochure in order to collect and to tabulate the experiences in abdominal surgery of a group of doctors over a period of thirty months at the front. The thought being to offer a standard of comparison for other doctors called to treat those wounded in the abdomen. He has embodied much of the available treatment of abdominal injuries founded on the war practice of this group of surgeons working under different conditions in different hospitals. Anyone who wishes to study the conditions met with during a period of thirty months at the front will find Dr. Wallace's book to be a rich fund of information covering new and complex experiences, to be well digested and to have carefully classified the work of many surgeons.

To read the book gives one pleasure; from the typographical point of view it is up to the highest standard.

---

**The Medical Record Visiting List,** the familiar pocket red book has appeared for 1919. Price for 30, 60 and 90 patients monthly, $1.50, $1.75 and $2.25 respectively. Publishers Wm. Wood & Co., 51 Fifth Ave., N. Y. The middle one used to be ample for us but in military work, we have often seen 100 or more patients a day and nearly or quite 1000 different men in a month.

